# Supplementary material for: CDK1–cyclin-B1-induced kindlin degradation drives focal adhesion disassembly at mitotic entry
Source: Nat Cell Biol. 2022 Apr 25;24(5):723–36. doi: 10.1038/s41556-022-00886-z (PMC9106588; doi:10.1038/s41556-022-00886-z)
Supplement: Supplementary file 1 — Reporting Summary [file 41556_2022_886_MOESM1_ESM.pdf]

nature portfolio

Corresponding author(s): Reinhard Fässler, Nan-Peng Chen

Last updated by author(s): Feb 26, 2022

Reporting Summary

Nature Portfolio wishes to improve the reproducibility of the work that we publish. This form provides structure for consistency and transparency in reporting. For further information on Nature Portfolio policies, see our Editorial Policies and the Editorial Policy Checklist.

Statistics

For all statistical analyses, confirm that the following items are present in the figure legend, table legend, main text, or Methods section.

n/a	Confirmed	
☐	☒	The exact sample size (*n*) for each experimental group/condition, given as a discrete number and unit of measurement
☐	☒	A statement on whether measurements were taken from distinct samples or whether the same sample was measured repeatedly
☐	☒	The statistical test(s) used AND whether they are one- or two-sided<br>*Only common tests should be described solely by name; describe more complex techniques in the Methods section.*
☒	☐	A description of all covariates tested
☐	☒	A description of any assumptions or corrections, such as tests of normality and adjustment for multiple comparisons
☐	☒	A full description of the statistical parameters including central tendency (e.g. means) or other basic estimates (e.g. regression coefficient) AND variation (e.g. standard deviation) or associated estimates of uncertainty (e.g. confidence intervals)
☐	☒	For null hypothesis testing, the test statistic (e.g. *F*, *t*, *r*) with confidence intervals, effect sizes, degrees of freedom and *P* value noted<br>*Give P values as exact values whenever suitable.*
☒	☐	For Bayesian analysis, information on the choice of priors and Markov chain Monte Carlo settings
☒	☐	For hierarchical and complex designs, identification of the appropriate level for tests and full reporting of outcomes
☒	☐	Estimates of effect sizes (e.g. Cohen's *d*, Pearson's *r*), indicating how they were calculated

Our web collection on statistics for biologists contains articles on many of the points above.

Software and code

Policy information about availability of computer code

Data collection	ZEN (Zeiss) for image collection on LSM780 confocal microscope (version 2.3 SP1 FP3, black) and SR1 Elyra PS.1 super-resolution microscope (version 2.1 SP1 FP3, black).<br>Visisystem (Visitron Systems) for image collection on wide field and TIRF microscope (Zeiss).<br>EVOS AutoFL system (Invitrogene) for image collection on EVOS live-cell microscope.<br>Harmony 4.9(Perkin Elmer) for image collection on Opera Phenix Plus High-Content Screening System.<br>FACsDIVA (BD, version 9.0) for data collection on LSRFortessa™ X-20 Cell Analyzer.<br>ImageQuant LAS 4000 for image collection of western blots on Fujifim LAS-4000<br>SoftMax Pro 7.1 for data collection for MTT and quantification of protein concentration on SpectraMax ABS Plus.
Data analysis	GraphPad Prism (version 6.0) was used for statistical analysis.<br>Flowjo (version 8.0) was used to analyze FACs data.<br>Fiji (Image J) was used for quantification of immunoblot bands, immunofluorescence and live-cell imaging signals.<br>Harmony (version 4.9) was used for quantification of high-through put siRNA screening.<br>Maxquant (version 1.6.7.0) was used for processing raw proteomic data.<br>Perseus software (version 1.5.5.3) was used to analyze proteomic data.

For manuscripts utilizing custom algorithms or software that are central to the research but not yet described in published literature, software must be made available to editors and reviewers. We strongly encourage code deposition in a community repository (e.g. GitHub). See the Nature Portfolio guidelines for submitting code & software for further information.

Data

Policy information about availability of data

All manuscripts must include a data availability statement. This statement should provide the following information, where applicable:

- Accession codes, unique identifiers, or web links for publicly available datasets
- A description of any restrictions on data availability
- For clinical datasets or third party data, please ensure that the statement adheres to our policy

Raw data of the proteomic analysis that support the findings of this study have been deposited in ProteomeXchange Consortium repository under Accession number PXD031829. Uncropped blots and statistical data of Fig. 1e; 2h; 3h; 4b, d, g, h, i, k, l; 5b, c, f; 6b, d, f; 7c, d, e and Extended Data Fig. 1c; 3c, d; 4c, e, f, h, k, n; 5b, d, f, h, i, k; 6e, g, h are provided in the source data. All other data supporting the finding of this study are available from the corresponding author on request.

Field-specific reporting

Please select the one below that is the best fit for your research. If you are not sure, read the appropriate sections before making your selection.

☒ Life sciences ☐ Behavioural & social sciences ☐ Ecological, evolutionary & environmental sciences

For a reference copy of the document with all sections, see nature.com/documents/nr-reporting-summary-flat.pdf

Life sciences study design

All studies must disclose on these points even when the disclosure is negative.

Sample size

The sample sizes were determined based on experience on the reliable measurement of the experimental results according to the standards in previous publications (Stewart et al, Nature, 2011; Lancaster et al, Dev Cell, 2013; Jones et al, JCB, 2018; Monster et al, JCB, 2021) in the field and sufficient to represent the significance of difference in different conditions. No statistical method was used to predetermine the sample size.

Data exclusions

No data was excluded in this study.

Replication

The sample size of each experiment and the number of experimental replicates were indicated in figure legends or methods. All experiments were successfully replicated.

Randomization

Samples were allocated into groups according to the kindlin 2 mutation the cell line carried, or based on the cell cycle stages revealed by phalloidin and DAPI staining, or based on the different siRNA used in the transfection. Data for comparisons of rounding speed, spindle orientation, retraction fiber length and number, focal adhesion number and size, DNA double strand break and duration of mitotic checkpoint in different kindlin mutant fibroblasts or upon different siRNA depletion were acquired by randomly taken images of cells in the well or on the coverslips. Statistical data for quantification was collected over months or years, thus ensure reproducibility despite being gathered from different passages.

Blinding

Image acquisition from different cell lines or at different conditions were not blinded but relied on unbiased data collection from random regions in the wells or coverslips.

Reporting for specific materials, systems and methods

We require information from authors about some types of materials, experimental systems and methods used in many studies. Here, indicate whether each material, system or method listed is relevant to your study. If you are not sure if a list item applies to your research, read the appropriate section before selecting a response.

Materials & experimental systems

n/a	Involved in the study	
☐	☒	Antibodies
☐	☒	Eukaryotic cell lines
☒	☐	Palaeontology and archaeology
☒	☐	Animals and other organisms
☒	☐	Human research participants
☒	☐	Clinical data
☒	☐	Dual use research of concern

Methods

n/a	Involved in the study	
☒	☐	ChIP-seq
☐	☒	Flow cytometry
☒	☐	MRI-based neuroimaging

Antibodies

Antibodies used

The following antibodies were used for western blotting (WB), immunofluorescence (IF) and flow cytometry (FC): kindlin-2 (clone

Antibodies used	3A3, MAB2617, mouse, Merck Millipore, 1:1000 for WB; 1:200 for IF); kindlin-2-pS181 (home-made, 1:5000 for WB; 1:400 for IF); kindlin-1 (home-made35, 1:1000 for WB); talin1 (T3287, Sigma, 1:1000 for WB); ILK (3856, rabbit, Cell signaling, 1:1000 for WB); PINCH (612710, mouse, Transduction Laboratories, 1:1000 for WB); vinculin (sc-5573, rabbit, Santa Cruz, 1:1000 for WB; V9131, Sigma, 1:500 for IF); FAK (3285, rabbit, cell signaling, 1:1000 for WB); paxillin (610051, mouse, Transduction Laboratories, 1:1000 for WB, 1:300 for IF); β1 integrin (9699, rabbit, cell signaling, 1:1000 for WB; TS2/16, mouse, Biolegend, 1:200 for IF; 12G10, MAB2247, mouse, Merck Millipore, 1:200 for IF; clone HMbeta1-1, 102207, hamster, BioLegend, 1:200 for FC); β3 integrin (M109-3, rat, Biozol, 1:200 for IF; clone VI-PL02, 336406, mouse, Biolegend, 1:200 for FC); β5 integrin (3629, rabbit, cell signaling, 1:200 for IF. ALULA, gift from Dr. Dean Sheppard, 1:200 for IF; clone KN52, 12-0497, mouse, ebioscience, 1:200 for FC); α5 integrin (clone 5H10-27, 557447, rat, PharMingen, 1:200 for FC); αV integrin (clone NKI-M9, 327909, mouse, Biolegend, 1:200 for FC); zyxin (clone 164D4, 307011, mouse, Synaptic system, 1:200 for IF); phospho-histone H3 (53348, rabbit, cell signaling, 1:1000 for WB; 1:500 for IF); cyclin A2 (4656, mouse, cell signaling, 1:1000 for WB); cyclin B1 (4138, rabbit, cell signaling, 1:1000 for WB); cyclin D1 (2978, rabbit, cell signaling, 1:1000 for WB); cyclin E2 (4132, rabbit, cell signaling, 1:1000 for WB); GAPDH (CB1001, mouse, Calbiochem, 1:10000 for WB); α-tubulin (2144, rabbit, cell signaling, 1:100 for IF); γ-tubulin (T6557, mouse, Sigma, 1:500 for IF); cullin9 (A300-098A, rabbit, bethyl, 1:1000 for WB; 1:200 for IF); FBXL10 (09-864, rabbit, Merck Millipore, 1:1000 for WB; 1:200 for IF); pS139-γH2AX (clone JBW301, 05-636, mouse, Millipore, 1:200 for IF); Mad2 (A300-301A, rabbit, Bethyl Laboratories, 1:200 for IF); hemagglutin (HA)-tag (rat, Roche, 1:1000 for WB); GFP (A10262, chicken IgY, Invitrogen, 1:1000 for WB); GFP-Booster Alexa Fluor 488 (gb2AF488, Chromotek, 1:500 for IF).
Validation	The application and dilution are listed above. The references of the used antibodies are the following. kindlin-2 (MAB2617, mouse, Merck Millipore, Theodosiou et al, eLife 2016. WB and IF); talin1 (T3287, Sigma, Theodosiou et al, eLife 2016; WB); ILK (3856, rabbit, Cell signaling, Hussain et al, J Neurosci. 2017. WB and IF); PINCH (612710, mouse, Transduction Laboratories, Donthamsetty et al, PLoS ONE. 2013. WB); vinculin (sc-5573, rabbit, Santa Cruz, O'Connor et al, Cancer research, 2013; V9131, Sigma, Feliciano et al, Nat Commun. 2021. WB and IF); FAK (3285, rabbit, cell signaling, Lundby et al, Cell. 2019. WB); paxillin (610051, mouse, Transduction Laboratories, Freeman et al, Science. 2020; WB and IF); β1 integrin (9699, rabbit, cell signaling, Matsumura et al, Nat Commun. 2016. WB; TS2/16, mouse, Biolegend, Freeman et al, Science. 2020. IF; 12G10, MAB2247, mouse, Merck Millipore, Nader et al, Nat Cell Biol. 2016. IF; clone HMbeta1-1, 102207, hamster, BioLegend, Baker et al, PNAS. 2012. FC); β3 integrin (336406, mouse, Biolegend, Hartmann et al, Cell Rep. 2019. FC); β5 integrin (clone KN52, 12-0497, mouse, ebioscience, Hansen et al, Nat Commun. 2016. FC); α5 integrin (clone 5H10-27, 557447, rat, PharMingen, Kharbili et al, Oncotarget. 2017. FC); αV integrin (clone NKI-M9, 327909, mouse, Biolegend, Debnath et al, Nature. 2018. FC); zyxin (clone 164D4, mouse, Synaptic system, Gill et al, EMBO J. 2015. WB); phospho-histone H3 (53348, rabbit, cell signaling, Ramos et al, Dev Cell, 2020. WB); cyclin A2 (4656, mouse, cell signaling, Bönisch et al, Nat Commun. 2017. WB); cyclin B1 (4138, rabbit, cell signaling, Kanakkanthara et al, Science. 2016. WB); cyclin D1 (2978, rabbit, cell signaling, Zhang et al, Nature. 2018. WB); cyclin E2 (4132, rabbit, cell signaling, Guo et al, Nat Cell Biol. 2016. WB); GAPDH (CB1001, mouse, Calbiochem, Gersch et al, Mol Cell. 2019: WB); α-tubulin (2144, rabbit, cell signaling, Bae et al, J Cell Biol. 2019. IF); γ-tubulin (T6557, mouse, Sigma, Douanne et al, Cell Rep. 2019: IF); cullin9 (A300-098A, rabbit, bethyl, Polajnar et al, EMBO Reports. 2017.WB); FBXL10 (09-864, rabbit, Merck Millipore, Han et al, Oncogene, 2016. WB); pS139-γH2AX (clone JBW301, 05-636, mouse, Millipore, Vermeij et al, Nature, 2016. WB and IF); Mad2 (A300-301A, rabbit, Bethyl Laboratories, Baron et al, eLife. 2016. IF); hemagglutin (HA)-tag (rat, Roche, Amodeo et al, J Cell Sci. 2018. WB); GFP (A10262, chicken IgY, Invitrogen, D'Souza et al, eLife. 2020. WB); GFP-Booster Alexa Fluor 488 (gb2AF488, Chromotek. Buchfellner et al, Plos One. 2016. IF).

Eukaryotic cell lines

Policy information about cell lines

Cell line source(s)	HeLa cell ATCC number: CCL-2<br>HEK293T cell ATCC number: CRL-3216<br>U2OS cell ATCC number: HTB-96<br>RPE1 cell ATCC number: CRL-4000<br>HAP1 cell: Horizon Discovery C859<br>Mouse K1/K2 double floxed fibroblast: generated in the lab
Authentication	HeLa, HEK293T, U2OS, RPE1 cell lines were purchased from ATCC and not authenticated in the study. HAP1 cells were purchased from Horizon Discovery and not authenticated in the lab. Mouse K1/K2 double floxed fibroblast was generate in our lab and previously described in eLife 2016;5:e10130.
Mycoplasma contamination	All cell lines used were tested negative for mycoplasma contamination
Commonly misidentified lines (See ICLAC register)	Not present in this study

Flow Cytometry

Plots

Confirm that:

☒ The axis labels state the marker and fluorochrome used (e.g. CD4-FITC).

☐ The axis scales are clearly visible. Include numbers along axes only for bottom left plot of group (a 'group' is an analysis of identical markers).

☒ All plots are contour plots with outliers or pseudocolor plots.

☒ A numerical value for number of cells or percentage (with statistics) is provided.

Methodology

Sample preparation	Interphase cells were trypsinized and mitotic cells were harvested by shake-off. For surface integrin profiling, cells were washed with cold PBS twice and incubated in 200 µL antibody solution (PBS with 2% BSA) for 1 hr on ice in dark. Cells were washed once with cold PBS and applied on LSRFortessa™ X-20 Cell Analyzer. For DNA content analysis, cells were fixed with 70% ethanol for 3 hr at -20 °C, washed twice with cold PBS and resuspended in 200 µL FxCycle PI/RNase Staining Solution, incubated for 20 min in dark and applied on LSRFortessa™ X-20 Cell Analyzer. Data was analysis with Flowjo software.
Instrument	LSRFortessa™ X-20 Cell Analyzer (BD biosciences)
Software	FACsDIVA (version 9.0) and Flowjo (version 8.0) software
Cell population abundance	Ten thousand events were recorded in each analysis. The whole population of harvested cells were stained and analyzed. The purity of the cells in different cell cycle phases were determined by DNA content analysis and immunoblotting of cyclins as described in the method section.
Gating strategy	No gating strategy was used.

☐ Tick this box to confirm that a figure exemplifying the gating strategy is provided in the Supplementary Information.
