# Supplementary figures and images for: CDK1–cyclin-B1-induced kindlin degradation drives focal adhesion disassembly at mitotic entry
Source: Nat Cell Biol. 2022 Apr 25;24(5):723–36. doi: 10.1038/s41556-022-00886-z (PMC9106588; doi:10.1038/s41556-022-00886-z)

Fig. 1a

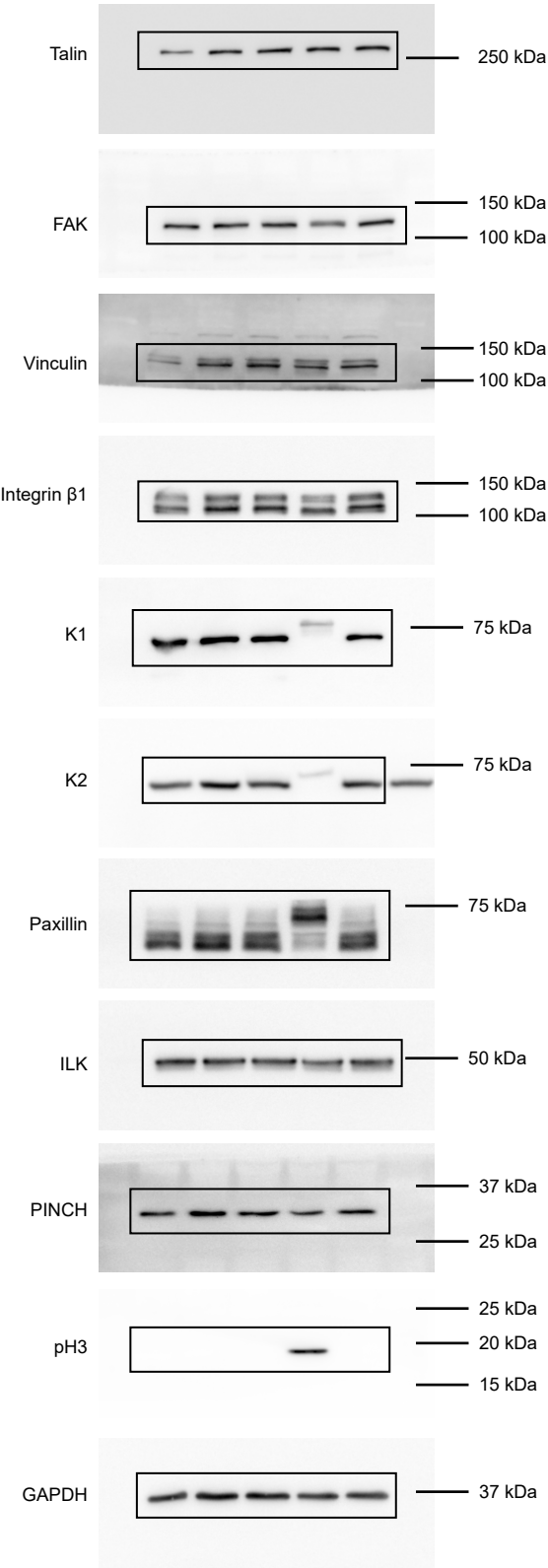

Fig. 1c

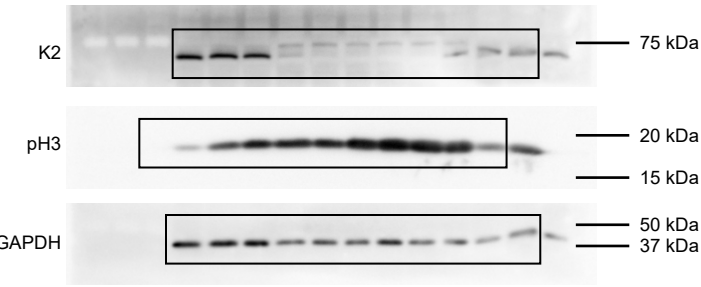

Supplement: Source Data Fig. 1 — Unprocessed western blots for Fig. 1. [file 41556_2022_886_MOESM10_ESM.pdf]

Fig. 3c

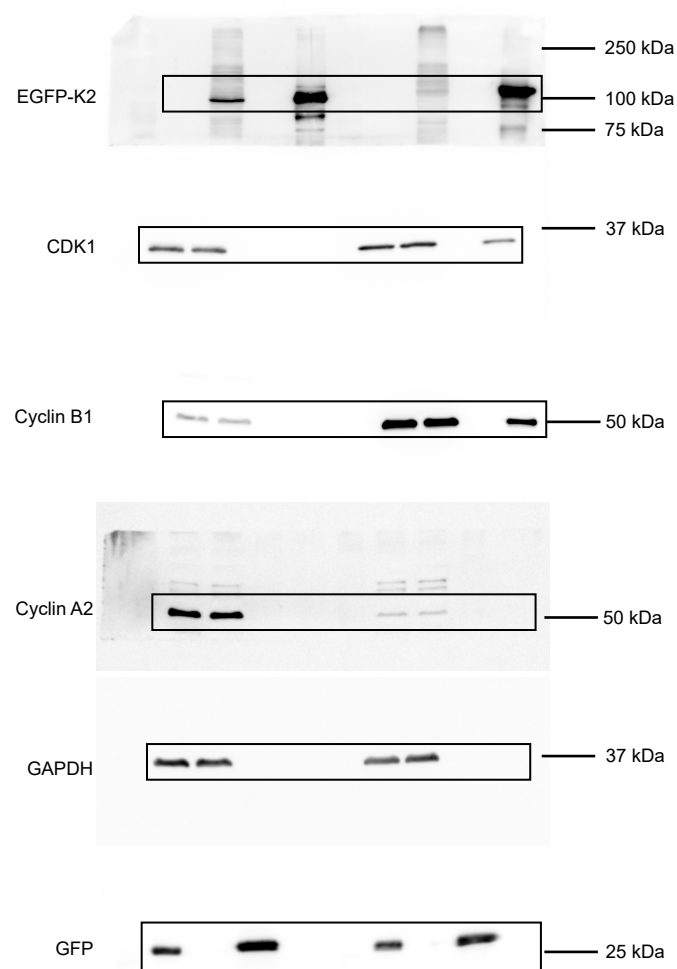

Fig. 3d

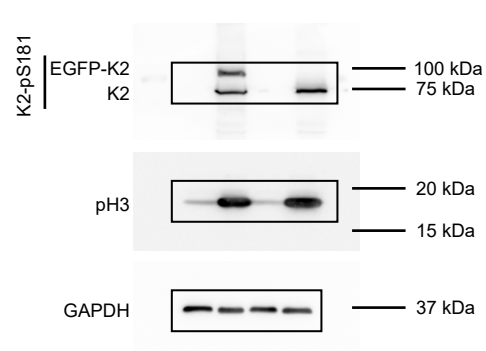

Fig. 3e

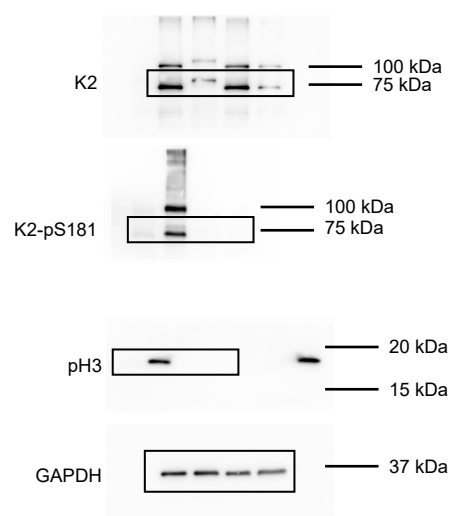

Supplement: Source Data Fig. 3 — Unprocessed western blots for Fig. 3. [file 41556_2022_886_MOESM13_ESM.pdf]

Fig. 4a

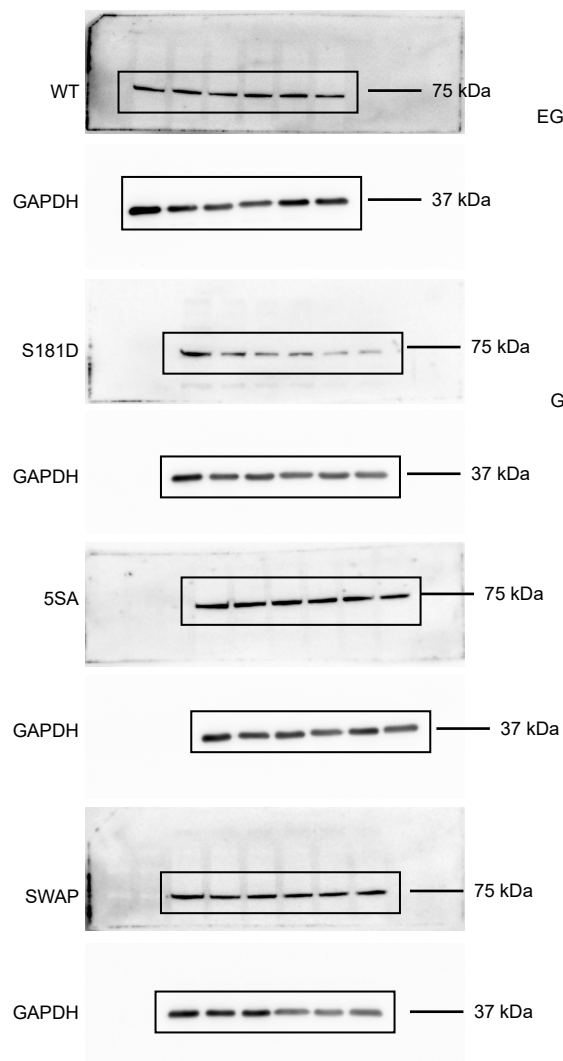

Fig. 4c

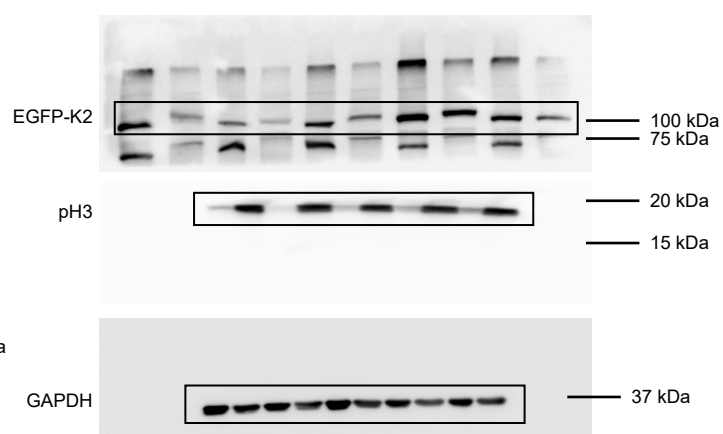

Supplement: Source Data Fig. 4 — Unprocessed western blots for Fig. 4. [file 41556_2022_886_MOESM15_ESM.pdf]

Fig. 6c

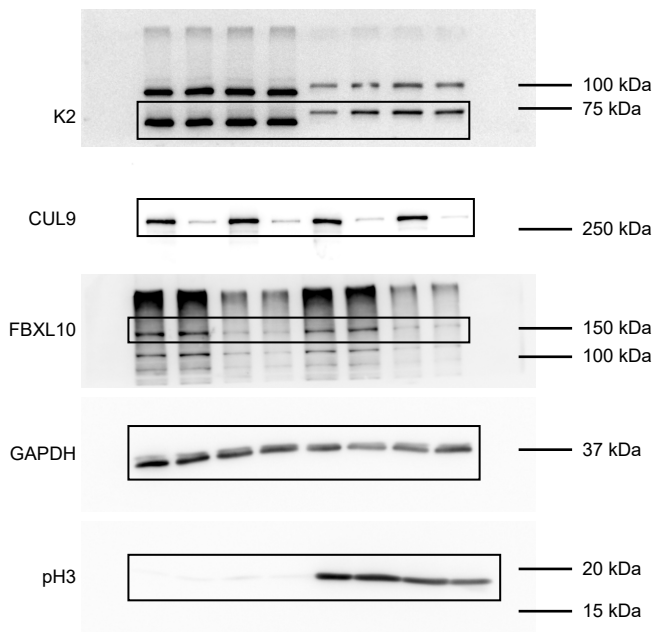

Fig. 6e

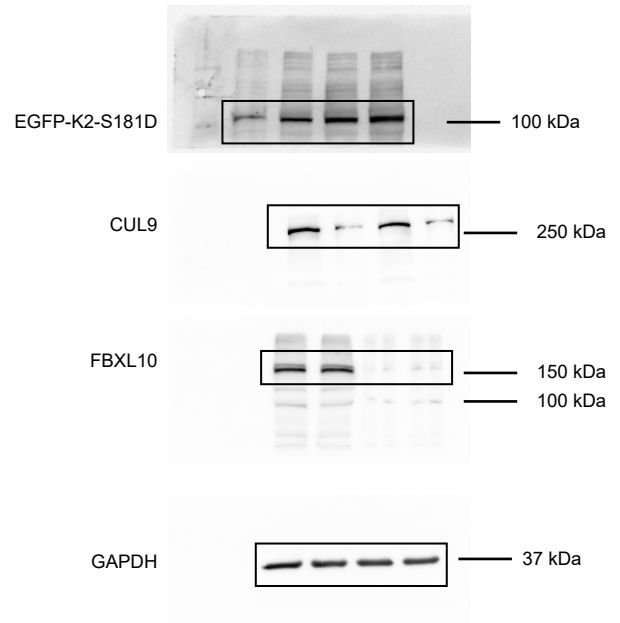

Fig. 6g

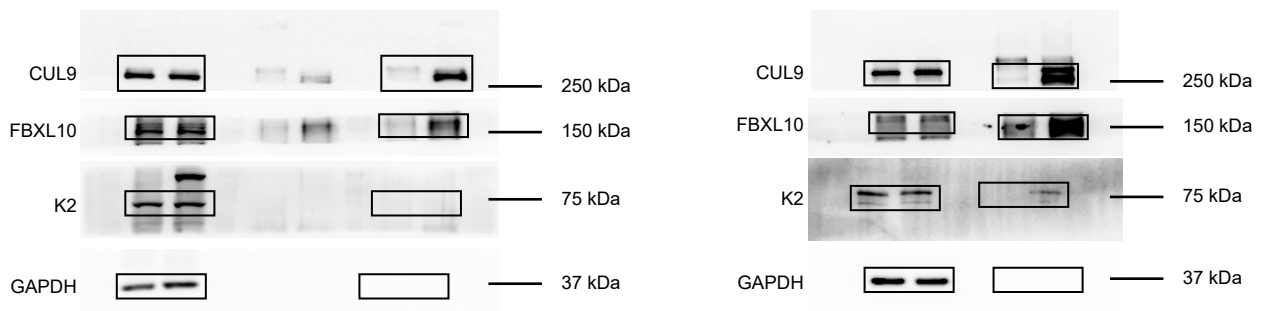

Fig. 6h

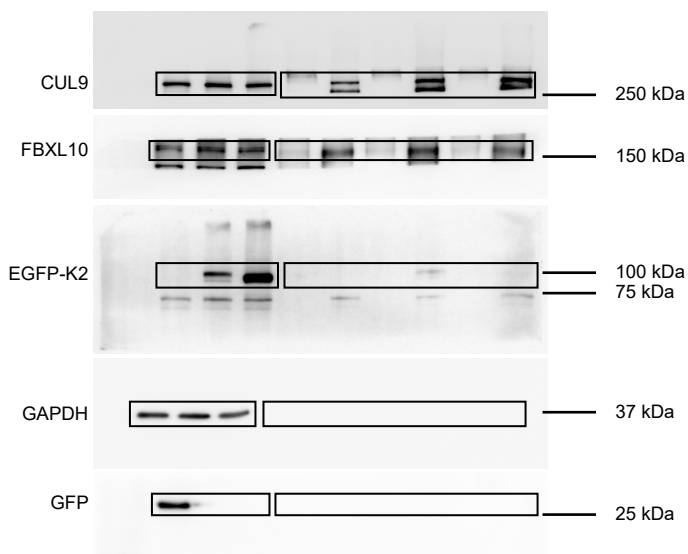

Supplement: Source Data Fig. 6 — Unprocessed western blots for Fig. 6. [file 41556_2022_886_MOESM18_ESM.pdf]

Fig. 7a

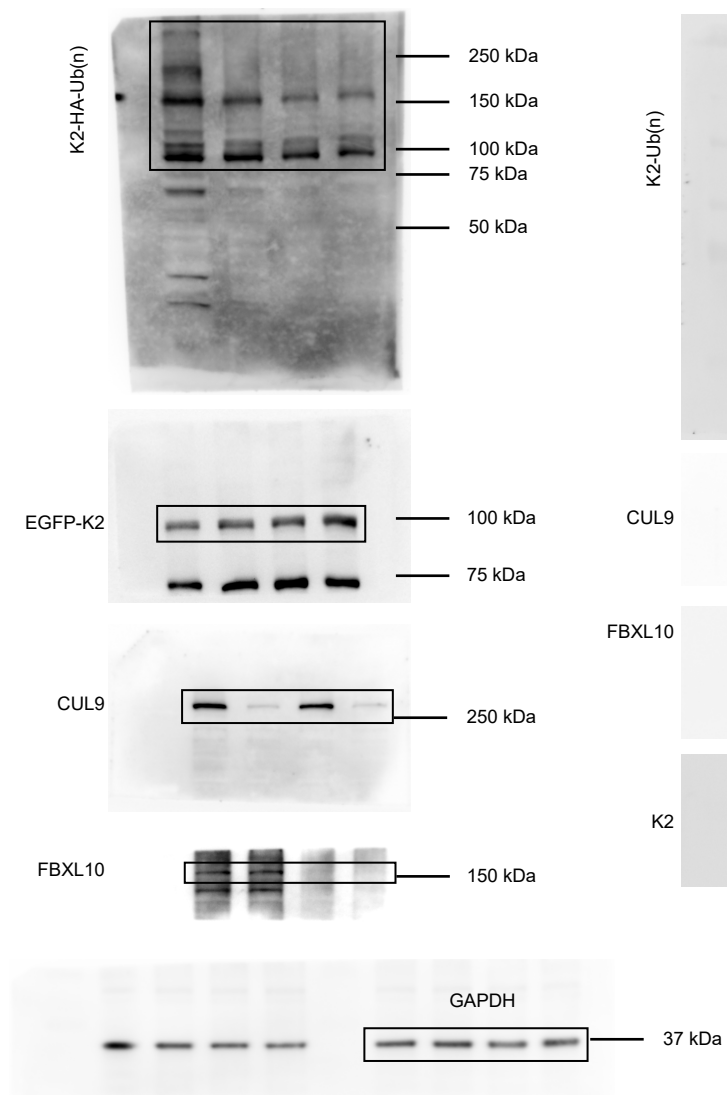

Fig. 7b

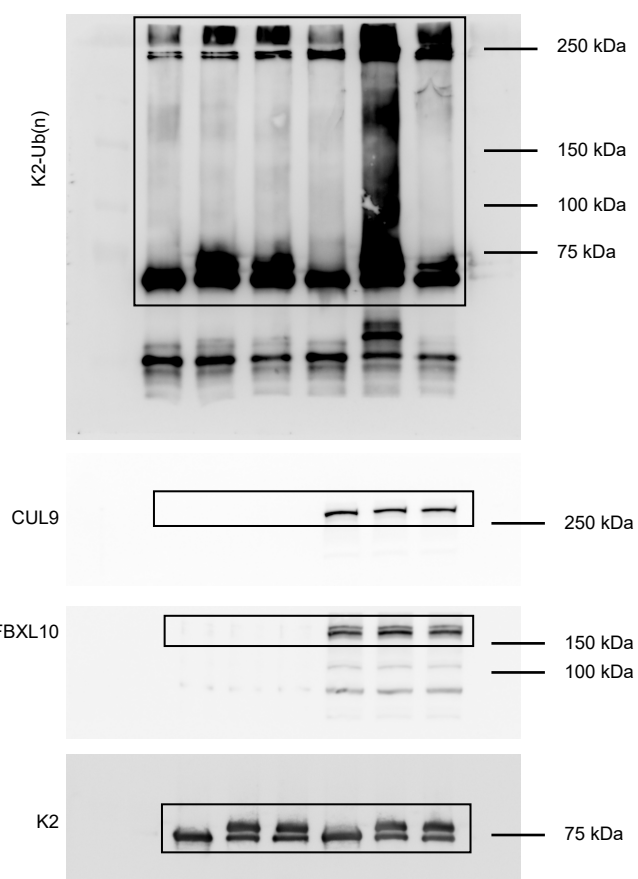

Supplement: Source Data Fig. 7 — Unprocessed western blots for Fig. 7. [file 41556_2022_886_MOESM20_ESM.pdf]

Extended Data Fig. 3a

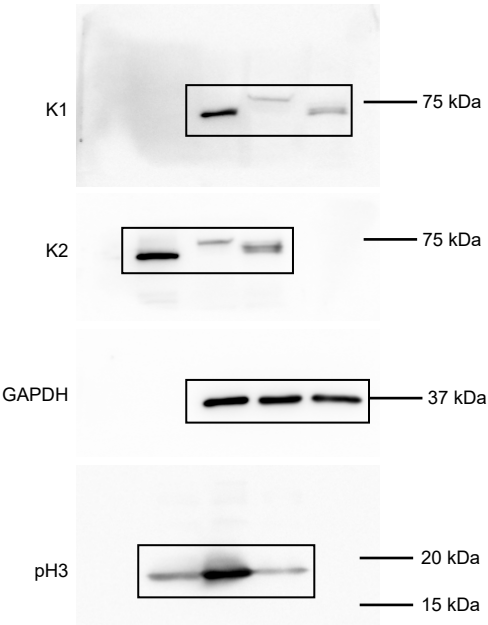

Supplement: Source Data Extended Data Fig. 3 — Unprocessed western blots for Extended Data Fig. 3. [file 41556_2022_886_MOESM24_ESM.pdf]

Extended Data Fig. 4b

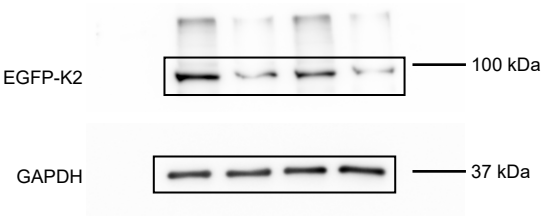

Extended Data Fig. 4g

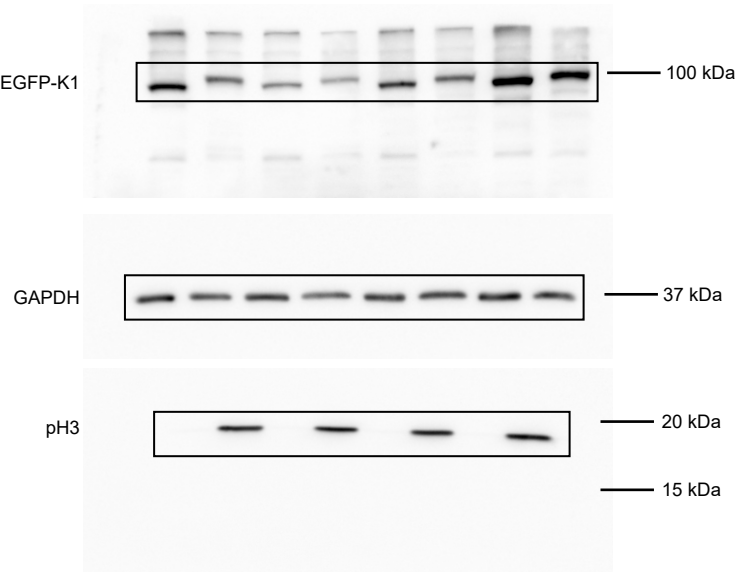

Extended Data Fig. 4i

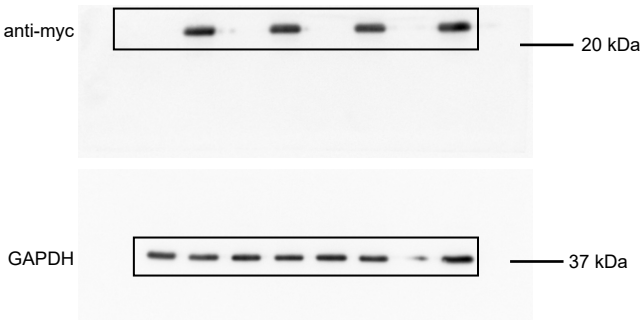

Extended Data Fig. 4l

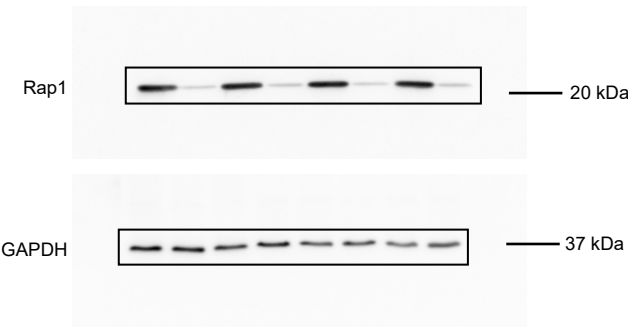

Supplement: Source Data Extended Data Fig. 4 — Unprocessed western blots for Extended Data Fig. 4. [file 41556_2022_886_MOESM26_ESM.pdf]

Extended Data Fig. 6b

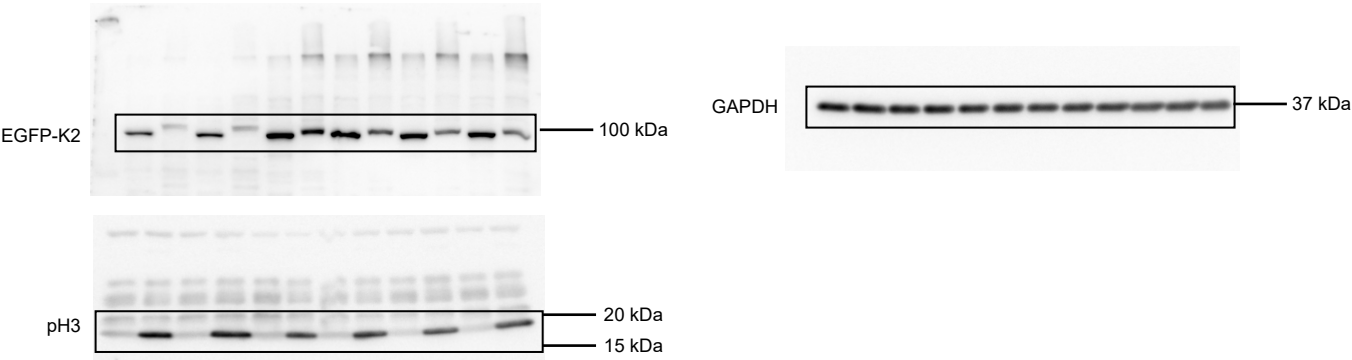

Supplement: Source Data Extended Data Fig. 6 — Unprocessed western blots for Extended Data Fig. 6. [file 41556_2022_886_MOESM29_ESM.pdf]
