# Supplementary material for: CDK1–cyclin-B1-induced kindlin degradation drives focal adhesion disassembly at mitotic entry
Source: Nat Cell Biol. 2022 Apr 25;24(5):723–36. doi: 10.1038/s41556-022-00886-z (PMC9106588; doi:10.1038/s41556-022-00886-z)

Extended Data Fig. 1a

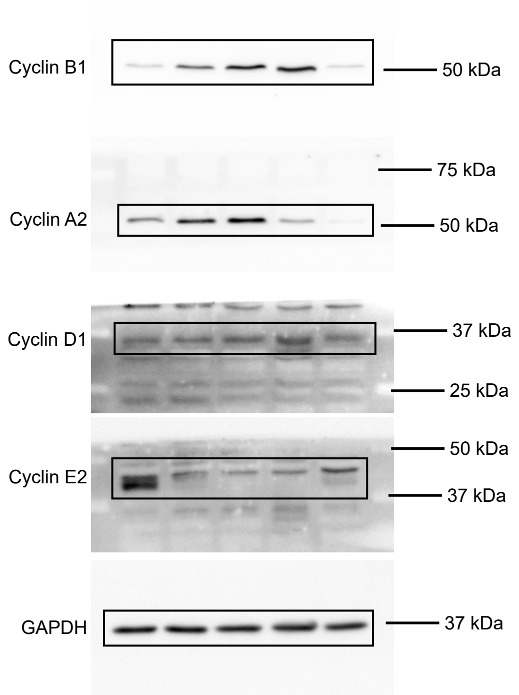

Extended Data Fig. 1e

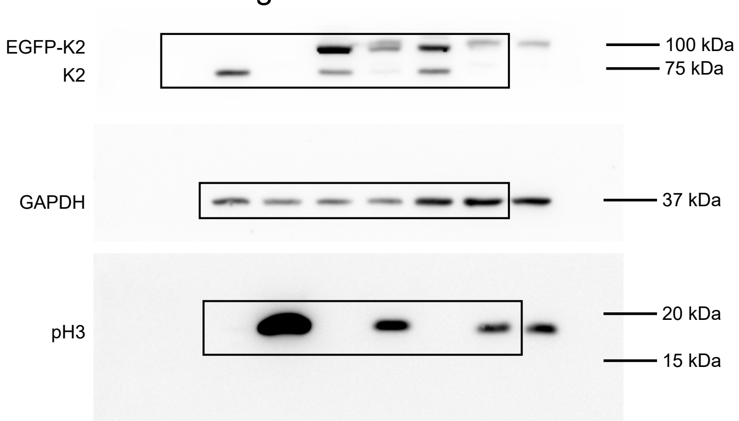

Extended Data Fig. 1f

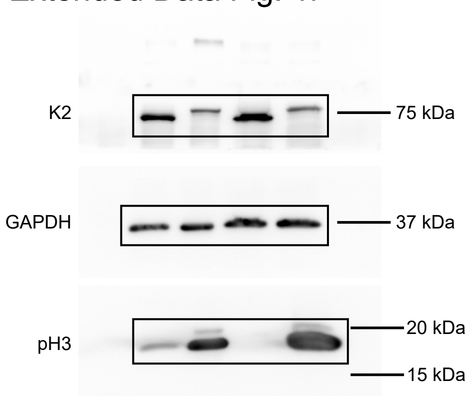

Extended Data Fig. 1g

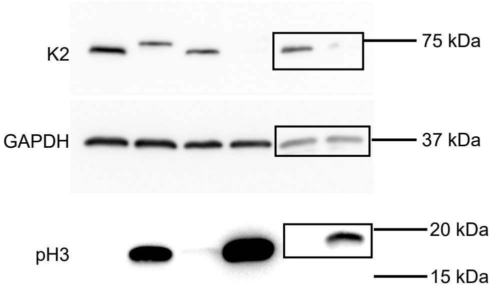

Extended Data Fig. 1h

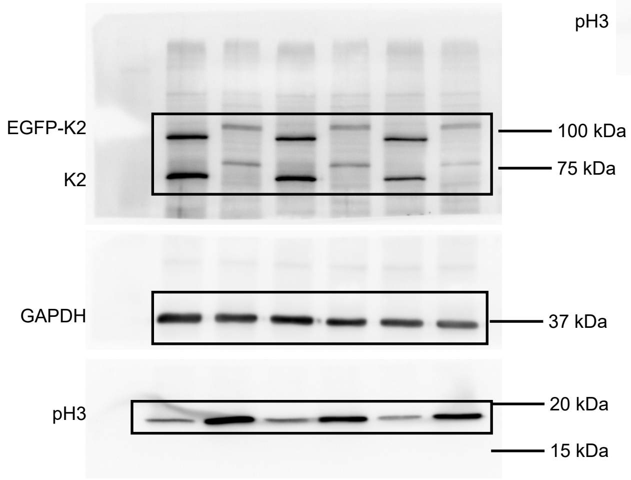

Extended Data Fig. 1i

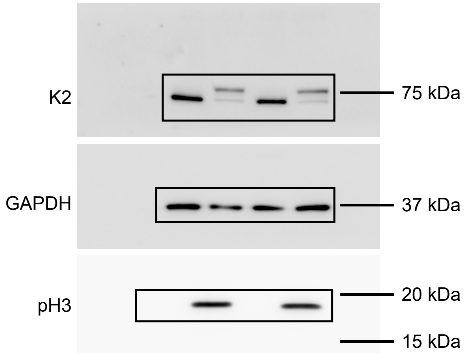

Extended Data Fig. 1j

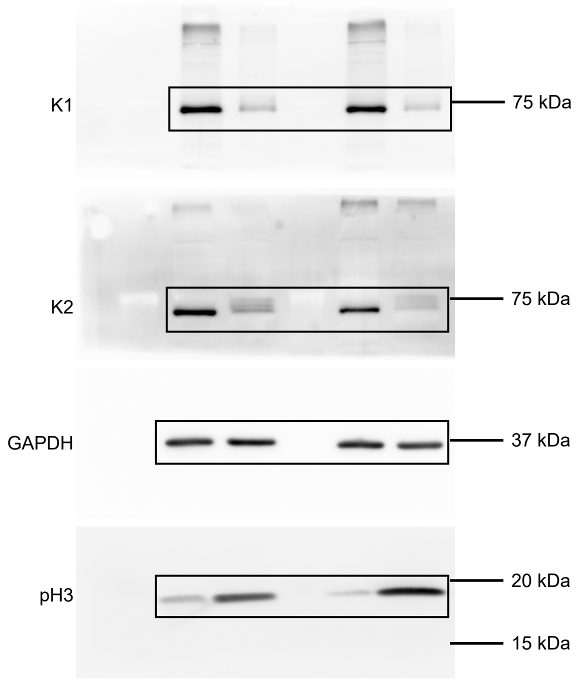

Extended Data Fig. 1k

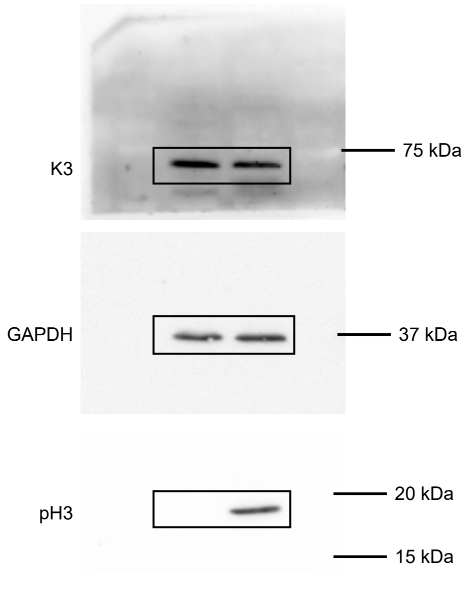

Supplement: Source Data Extended Data Fig. 1 — Unprocessed western blots for Extended Data Fig. 1. [file 41556_2022_886_MOESM22_ESM.pdf]
